# Predicting West Nile virus circulation: a 20-year spatiotemporal study in humans and animals in Spain, 2003 to 2022

**DOI:** 10.2807/1560-7917.ES.2026.31.16.2500535

**Published:** 2026-04-23

**Authors:** José-María García-Carrasco, Raimundo Real, Ignacio García-Bocanegra, Moisés Gonzálvez, David Cano-Terriza, Daniel Bravo-Barriga, Marina Segura, Jesús Olivero

**Affiliations:** 1 Department of Entomology, Washington State University, Pullman, Washington, United States; 2 Grupo de Biogeografía, Diversidad y Conservación, Departamento de Biología Animal, Facultad de Ciencias, Universidad de Málaga, Malaga, Spain; 3 Instituto IBYDA, Centro de Experimentación Grice-Hutchinson, Malaga, Spain; 4 Departamento de Sanidad Animal, Grupo de Investigación en Sanidad Animal y Zoonosis (GISAZ), UIC Zoonosis y Enfermedades Emergentes ENZOEM, Facultad de Veterinaria, Universidad de Córdoba, Córdoba, Spain; 5 CIBERINFEC, ISCIII CIBER de Enfermedades Infecciosas, Instituto de Salud Carlos III, Madrid, Spain; 6 Grupo Sanidad y Biotecnología (SaBio), Instituto de Investigación en Recursos Cinegéticos (IREC), CSIC-UCLM-JCCM, Ciudad Real, Spain; 7 Área de Parasitología y Enfermedades Parasitarias, Departamento de Sanidad Animal, Grupo de Investigación en Sanidad Animal y Zoonosis (GISAZ), UIC Zoonosis y Enfermedades Emergentes ENZOEM, Facultad de Veterinaria, Universidad de Córdoba, Córdoba, Spain; 8 International Vaccination Centre of Malaga, Maritime Port of Malaga, Ministry of Health, Consumption and Social Welfare, Government of Spain, Malaga, Spain

**Keywords:** Climate, Early warning system, One Health, Prediction, Risk, Surveillance, Zoonosis

## Abstract

**BACKGROUND:**

While West Nile virus (WNV), a mosquito-borne zoonotic pathogen, is detected every year in animals in Spain, clinical human cases occur more sporadically. Most explanatory and predictive models for WNV circulation focus on single components and aggregate multiple year data into a single dataset.

**AIM:**

We sought WNV circulation environmental drivers across different ecological components (vectors, reservoirs, and dead-end hosts), by analysing their spatial and temporal dynamics.

**METHODS:**

We used active and passive surveillance data collected in Spain between 2003 and 2022, encompassing mosquitoes, 120 bird species, 115 mammal species, and humans. To understand WNV circulation, mosquito spatial and host spatiotemporal models were developed, incorporating current and lagged environmental variables. Our One Health approach integrated the different models to determine WNV exposure risk, including 1 year in advance.

**RESULTS:**

Over 20 years, WNV exposure risk in Spain rose by 19% in birds, 17% in non-human mammals, and 38% in humans. In birds and non-human mammals, exposure more likely occurred in areas experiencing mean respective annual temperatures > 5 °C and > 8 °C in the previous year. In humans, increased exposure risk concurred with mild winters (> 5.3 °C). Integrating mosquito and host models found the country’s southern half and mediterranean coast most suited for WNV. Predictive models solely using prior-year variables yielded comparable results to contemporaneous ones.

**CONCLUSION:**

The models suggest that annual human WNV transmission may occur more regularly than evidenced by surveillance, possibly due to asymptomatic or misdiagnosed cases. Our framework could serve as an early warning tool, enhancing outbreak preparedness up to 1 year ahead.

**Key public health message**

**What did you want to address in this study and why?**

West Nile virus (WNV) is transmitted to bird and mammalian hosts by mosquito vectors. In Spain while WNV infections are observed annually in wildlife, sporadic human cases are increasing. Using Spanish surveillance data from 2003 to 2022, we developed spatiotemporal models to see how mosquitoes and environmental variables affect WNV circulation in the country. This allowed to assess WNV exposure risk in birds and mammals and to anticipate outbreaks.

**What have we learnt from this study?**

In birds and mammalian animals, WNV exposure risk in Spain increased by 17–19% over 20 years, probably because some areas experienced high mean annual temperatures. Human risk rose by 38%, with infections occurring in areas favourable for mosquitoes and exposure risk likely driven by mild winters. Combining human, animal, mosquito, and environmental data found the southern half of Spain best suited for WNV and could predict outbreaks up to 1 year in advance.

**What are the implications of your findings for public health?**

Improved ways to anticipate WNV outbreaks allows health authorities to timely control mosquitoes when environmental and epidemiological conditions favour WNV circulation, as recommended in European surveillance frameworks. Our integrated approach suggests that human cases occur every year but may go undetected due to mild or misdiagnosed infections. Ongoing comprehensive monitoring is thus important, even when no human cases are reported.

## Introduction

West Nile virus (WNV; genus *Orthoflavivirus,* family Flaviviridae) remains one of the most widely distributed and impactful mosquito-borne zoonotic viruses worldwide [1]. This virus is primarily maintained in an enzootic cycle involving birds as amplifying hosts and mosquitoes, predominantly of the genus *Culex,* as the main competent vectors [1,2]. Spillover events lead to infections in mammals, including humans, who serve as dead-end hosts due to insufficient viraemia levels to maintain the transmission cycle. While in people most infections are asymptomatic, approximately 20% of these cause symptoms ranging from mild fever to severe neurological disorders, with nearly 10% fatality among severe cases [3].

WNV was first detected in Europe in 1962 and has gradually spread across the continent, with Spain representing an important area of virus circulation and amplification during the transmission season (July–November) [4-9]. Even though the first human case in Spain was reported in 2004 [10], the virus had already been detected in animals (birds, horses and mosquitoes) by 2003 [6,7]. Although there were no human outbreaks for years in Spain, the virus persisted in animals, as shown by a sharp rise in WNV detections in birds, equids, and other mammals in 2020 [11]. Epidemics in humans occurred in 2010, 2016 and have continued since 2020, with a record-breaking outbreak in 2024 that had an unusually early onset and resulted in 158 human cases (including 20 fatalities) and broader geographic expansion within the country [8,12,13]. The recent increase in the frequency, intensity, and geographic spread of human cases highlights the urgent need to understand the environmental factors driving these spatial and temporal patterns.

Computational models have been widely employed to explain and predict the distribution of infectious diseases such as WNV by leveraging climatic [14], ecological [15], and socioeconomic data [16]. Typically, these models focus on specific components of the WNV transmission cycle, such as competent vectors [17,18], reservoirs [19,20], dead-end hosts [21,22], or, generally, human cases [23,24]. However, such partial approaches miss the interactions among vectors, reservoirs and dead-end hosts that operate through diverse WNV exposure pathways, highlighting the need for integrated active and passive surveillance — especially for a disease where over 80% of human infections are asymptomatic. In terms of temporal scale, models usually concentrate on specific outbreak years or, when analysing multiple years, aggregate all data into a single pool (see references above). This approach, while useful for identifying general trends, can overlook the interannual variability in WNV cases and fails to capture the dynamic nature of the virus's transmission over time.

By integrating multi-sectoral surveillance of vectors, reservoirs, and dead-end hosts over 20 years, this study aimed at developing spatiotemporal predictive models that capture the dynamic interactions between epidemiological, environmental and ecological factors, to anticipate WNV epidemic-prone areas in Spain up to 1 year in advance and support the timely implementation of preventive public health.

## Methods

### General approach

We adopt here a One Health approach, emphasising the interconnectedness of human and animal health with environmental components. The study was conducted through active interdisciplinary collaboration, involving biologists, veterinarians, entomologists, physicians, epidemiologists, and biogeographers, who jointly contributed to study design, data collection, analysis, and interpretation. This integrated surveillance approach is considered essential by European guidelines to identify potential public health risks and enable timely interventions.

### Data collection

We collected data from mainland Spain on vectors and some WNV-exposed hosts in order to map their locations between 2003 and 2022. 

The occurrence of the main vectors of the virus in Europe, namely the genus *Culex,* was obtained from literature and a citizen science system (Mosquito Alert) [25-27]. These data reflect human-mosquito interactions and provide information about exposure risk. Information about mosquito distribution in Spain in 2003−2022 is described in Supplementary Figure S1.

Animal exposure to WNV was evaluated using serological tests (detection of anti-WNV antibodies (immunoglobulin (Ig)G and/or IgM) by ELISA and/or virus neutralisation tests), based on both active (derived from systematic monitoring programmes) and passive surveillance data (obtained from the reporting of spontaneous or clinically suspected cases) collected from official sources and previous studies [11,28-41]. The dataset comprised 37 mammal families (115 species) and 50 bird families (120 species), including wild, zoo-kept, and domesticated animals, with more detailed information provided in Supplementary Table S1. Between 2003 and 2022, the annual distributions of animals exposed to WNV (or not) in Spain, as found by seroprevalence investigations, are illustrated in Supplementary Figure S2 for birds and in Supplementary Figure S3 for non-human mammals.

Data on clinical human cases were provided by the National Centre for Epidemiology, Carlos III Health Institute, and defined in accordance with the national surveillance protocol, as any person with compatible neurological symptoms or signs (encephalitis, viral meningitis, Guillain–Barré syndrome and acute flaccid paralysis) whether or not accompanied by fever (> 38.5 °C) and at least one of the following laboratory criteria: (i) isolation of WNV, (ii) presence of IgM antibodies in a cerebrospinal fluid sample, (iii) detection of WNV RNA by reverse transcription (RT)-PCR, or (iv) a specific antibody response (increased IgM levels together with the detection of specific IgG antibodies by neutralisation assay) [42]. The distribution of WNV clinical cases each year in the 2003 to 2022 period is shown in Supplementary Figure S4.

### Spatial and spatiotemporal units and models

Locations of mosquitoes and virus exposure in birds and mammals were assigned to municipalities, which served as Operational Geographic Units (OGUs). These OGUs facilitate within their boundaries the collection of environmental data that may influence case distribution, while also helping to reduce spatial autocorrelation. 

While we used OGUs for spatial models, we used Operational Spatio-Temporal Units (OSTUs) for spatiotemporal models. OSTUs represent OGUs within a specific year *t* that, in our case, corresponded with a hydro-meteorological year (see below). That is, each spatial model included 8,141 OGUs (i.e. municipalities), while each spatiotemporal model comprised 162,820 OSTUs (i.e. 8,141 municipalities over 20 years of study). 

We employed spatial models (and OGUs) for the mosquito data to identify areas most suitable for WNV vectors, whereas spatiotemporal models (and OSTUs) were used to assess risk areas over different years of WNV circulation in birds, non-human mammals, and humans.

### Environmental variables

We compiled both spatial and spatiotemporal variables, which are presented in Supplementary Table S2.

Spatial variables represent environmental features that, in our study period, can be assumed to remain mostly constant over time but vary across space, such as topography, hydrography, and anthropogenic factors. In contrast, spatiotemporal variables show changes across both space and time, and include meteorological data as well as the Normalised Difference Vegetation Index (NDVI). The NDVI reflects vegetation density, habitat quality, and microclimatic conditions, all of which are critical for mosquito survival and breeding. 

Since our goal for the spatiotemporal models was to develop an explanatory framework while maximising their predictive potential, we included variables from both the year in which exposure was detected and the preceding year. In Spain, the epidemiology of cases exhibits a marked seasonality, with most cases concentrated between July and November [9]. To capture the maximum amount of temporally relevant information before the onset of cases, we employed hydro-meteorological years instead of calendar years. These years span the months from September to August, allowing the integration of climatic and environmental conditions that may influence infection dynamics. 

For the spatial model (mosquito model), we used spatial variables (topography, hydrography, and anthropogenic variables) and calculated the mean value of the spatiotemporal variables (meteorological and NDVI data) over the 20-year period to obtain long-term environmental averages for modelling vector favourability across municipalities. 

In contrast, for the spatiotemporal models (WNV circulation in birds, non-human mammals and humans), we used spatial (the previously cited variables plus mosquito model) and spatiotemporal variables.

### Model development

Before developing the different multivariate models, we assessed the relationship between each explanatory variable and the respective distribution of mosquitoes, and of other WNV-exposed animals/humans in each surveillance dataset (birds, non-human mammals and humans), using the Rao's score test, a statistical method that evaluates whether a variable contributes significantly to the model [43]. This initial assessment was performed within a binary logistic regression framework. The logistic regression was subsequently implemented as a supervised machine learning classifier [44]. This implementation involved training the model on labelled data and optimising the model coefficients to minimise the prediction error, which yielded the final prediction models.

We assessed multicollinearity among the variables through Spearman correlation analysis. Pairs of variables with a correlation coefficient exceeding 0.8 were identified, and the less informative variable — determined by the significance of Rao's score test — was excluded [43]. Given the large number of variables, we controlled for the false discovery rate (FDR) [45] and retained only the significant variables according to Rao's score test under an FDR threshold of q < 0.05 for subsequent analyses.

Variables that passed these initial filters were then incorporated into ensemble models using multivariate stepwise logistic regression. In this approach, variables were added iteratively to an initial null model only if their inclusion significantly improved the regression.

Probability values (*P*) of occurrence of mosquitoes or WNV exposed mammals/birds obtained from logistic regression for a given OGU or OSTU were converted into favourability values (*F*) using the Favourability Function, which is mathematically formulated in Supplementary Equation S1. These values ranged from 0 to 1. This function transforms probabilities into measures of how much local conditions favour the presence of an event, making results comparable across events with different prevalence [46]. A *F* of 0.5 indicates that the local probability of detecting exposure to the virus is equal to the overall proportion of affected OGUs or OSTUs in Spain. An *F* value greater than 0.5 indicates that the local probability of occurrence is higher than the overall proportion, thus signifying increased risk. Conversely, an *F* value less than 0.5 indicates that the local probability is lower than the overall proportion, signifying decreased risk. Therefore, *F* values serve as a risk measure, representing the degree to which environmental conditions favour WNV exposure.

The Favourability Function adjusts for variation in event probability relative to its occurrence rate, making *F* values comparable across events of different frequencies [47]. This enables favourability models to be compared and combined using fuzzy set theory tools [22,48,49]. The models were developed, and favourability values for each OGU and OSTU were calculated, using the *fuzzySim* R package (v.4.9.9) [50].

### Model extrapolation

The animal spatiotemporal models were developed using data from municipalities actively sampled for WNV exposure, including both positive and negative cases and the year of exposure detection. By focusing on sampled areas (i.e. municipalities with confirmed positive and confirmed negative results), we avoid further generalisations that were based on unsampled areas, ensuring more robust and reliable predictions. The model identified the key spatiotemporal variables that best explain the distribution of WNV positive cases in the sampled municipalities (across the years) and determined the corresponding coefficient for each variable. The models were then extrapolated across territory and the full time series (2003–2022). This was achieved by projecting the model's coefficients onto the annual values of the environmental covariates for every single OSTU. In the extrapolation process we ensured that the predictor variable values did not exceed the ranges observed within the training area (sampled municipalities). The process generated an *F* for all OSTUs, identifying risk of exposure, even in not actively sampled OSTUs, based on their similar environmental conditions to sampled ones.

In contrast, for the human spatiotemporal models, since all reported human cases resulted from symptomatic individuals rather than from active infection screening campaigns, we considered detected infections as presence points and non-reported areas as absences.

### 1-year lag predictive modelling

Our spatiotemporal models originally included both contemporaneous variables (e.g. temperature, precipitation, NDVI in year *t* for cases recorded in year *t*) and variables from the preceding year (*t* − 1). To assess the models’ predictive capacity, we additionally developed a set of ‘1-year-ahead’ models in which all spatiotemporal predictors were replaced by their previous-year values (i.e. outcomes in year *t* were modelled using covariate values from year *t* − 1 only). This approach tests whether the models retain explanatory power when fed only data available 1 year earlier, i.e. formulation closer to operational forecasting. We reported and compared model performance metrics for contemporaneous and 1-year-lagged models.

### Model evaluation

A significant spatiotemporal model identifies favourable environmental conditions associated with the exposure that were consistent year after year and throughout the 20-year period. Because of this, instead of generating a single map showing favourable conditions across Spain, the models produce a volume comprising 20 maps, each representing favourable conditions across Spain for a specific year. We assessed the model's classification accuracy and discrimination ability over the occurrence or not of WNV cases using the *modEvA* R package [51].

We calculated sensitivity (i.e. the proportion of correctly classified presences), specificity (i.e. the proportion of correctly classified absences), underprediction (i.e. the proportion of OGUs or OSTUs considered as unfavourable that, however, coincide with presences), overprediction (i.e. the proportion of OGUs or OSTUs considered as favourable that nevertheless coincide with absences), correct classification rate (CCR, the proportion of correctly classified presences and absences), and the true skill statistic (TSS, as: sensitivity + specificity – 1), with *F =* 0.5 serving as the classification threshold [51,52] (which is equivalent to using prevalence in the assessment of probability). 

For sensitivity, specificity, and CCR, values range from 0 (no predictive power) to 1 (perfect predictive power). Conversely, for underprediction and overprediction, 0 represents the ideal model, indicating no under- or overprediction. Finally, TSS ranges from −1 to 1, with 1 representing perfect prediction, 0 random performance, and −1 indicating performance worse than random chance. The model's discrimination ability was evaluated using the area under the receiver operating characteristic curve (AUC, threshold-independent measure of discrimination (0.5–1)).

### Combination of risk models

We calculated the total risk in Spain for each year based on the cardinality of all its OSTUs (i.e. the sum of favourability values for all the municipalities *t* year). Since each spatiotemporal model (in birds, non-human mammals and humans) consists of a volume of 20 maps, one for each year, this allowed us to plot the overall increase in risk over the 20-year period. 

Additionally, the 20 maps of each model were combined into a single map using fuzzy intersection or fuzzy union, as formulated in Supplementary Equation S2. The fuzzy intersection selects, for each municipality, the minimum favourability value observed over the 20 years (F_2003_ ∩ F_2004_ ∩…∩ F_2022_), highlighting areas where risk has remained consistently high over the study period. In contrast, the fuzzy union, which selects the maximum values over the years (F_2003_ ∪ F_2004_ ∪…∪ F_2022_), highlights areas where risk was high in at least one of the study years. We applied fuzzy intersection to the animal models to emphasise areas of sustained high risk over time, whereas for the human models, we used fuzzy union to capture any occurrence of high risk over the years and to avoid overlooking at-risk areas, given the marked interannual fluctuations and under-reported human infections. 

Finally, the spatiotemporal models of risk of exposure in birds, non-human mammals and humans were combined with each other using the fuzzy union to identify risk areas for WNV circulation in at least one type of host. 

The combined output was intersected with the spatial mosquito model to identify areas where high-risk overlaps with favourable vector conditions.

## Results

### Risk models

For mosquitoes, the spatial model showed higher favourability in low-lying, densely human-populated areas with water presence, marked seasonal variation in precipitation, and high NDVI values indicating green vegetation during the driest seasons, as presented in Supplementary Table S3. This mainly corresponded to coastal regions near main rivers, as illustrated in Supplementary Figure S5.

**Figure 1:**
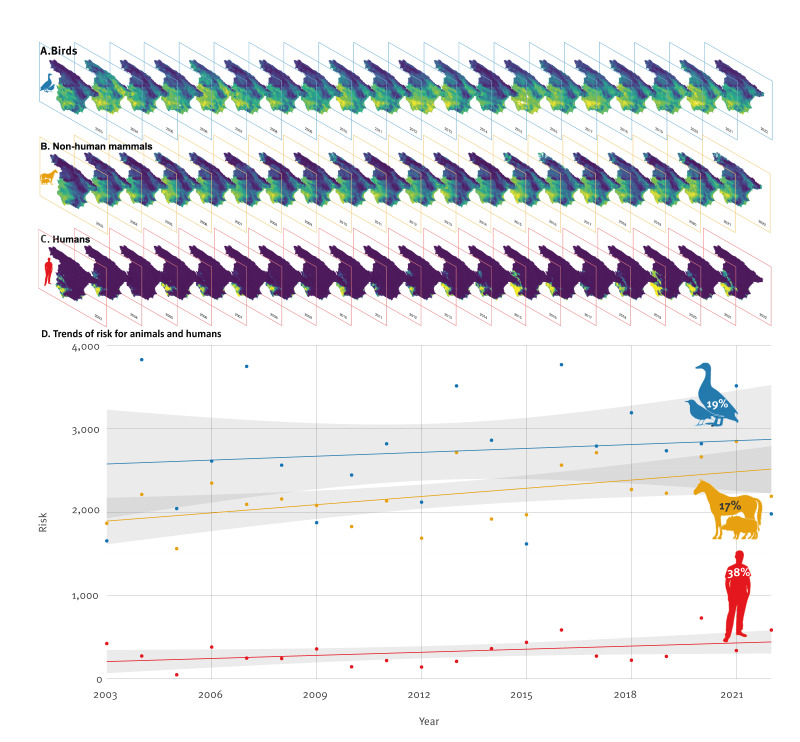
Change in the risk of West Nile virus circulation in the spatiotemporal models for (A) birds, (B) non-human mammals, and (C) humans, as well as (D) temporal trends of the total risk for birds, non-human mammals and humans, Spain, 2003–2022

Across birds, non-human mammals, and humans, the spatiotemporal models revealed distinct patterns of virus circulation from 2003 to 2022 (Figure 1.A-D). In birds, the risk of virus circulation was primarily observed in non-anthropised areas, far from human settlements, with high mean temperatures in the year preceding detection (16.5 ± 1.5 °C), comparatively moderate maximum temperatures in the current year (33.5 ± 2.5 °C) (Supplementary Table S3). The risk was widely distributed across Spain throughout the study period, with consistently high-risk in the southern half of the country. Years such as 2004, 2007, and 2016 showed an expansion of risk into the northern half of the country (Figure 1.A), leading to periods of highest overall risk (Figure 1.D). The risk of WNV exposure to birds increased 19% over the 20-year period.

The figure shows the risk for Spain overall. In panels A–C, the lighter colours on the maps indicate higher risk. Panel D depicts temporal trends of the total risk for birds (blue), non-human mammals (orange), and humans (red). Points represent total annual risk values, while solid lines show linear trends with shaded confidence intervals. Percentages indicate the increase of risk in the different groups over the 20 years.

In non-human mammals, virus circulation occurred in flat regions with high mean temperatures (17.1 ± 1.2 °C), low precipitation (553 ± 236mm), and high seasonality in rainfall (i.e. a strongly pronounced concentration of precipitation within certain months of the year), all of them during the previous year, combined with, in the current year, lower rainfall seasonality i.e. a uniform concentration of precipitation throughout the year (Supplementary Table S3). This meteorological scenario seems to align with vegetation dynamics, which also influence the risk of circulation. On the map, this translates into a consistent risk in the south-western region over the years, with expansion into the eastern and northern parts of the country in recent years (Figure 1.B). Over these 20 years, the risk of WNV exposure to non-human mammals rose by 17% (Figure 1.D).

In humans, infections were concentrated in areas favourable for mosquito presence, with high mean temperatures (18.3 ± 0.8 °C) and concurred with relatively mild winter conditions (minimum temperature of 5.3 ± 1.7 °C) (Supplementary Table S3). Rainfall pointed to the same risk scenario as in the model for non-human mammals, with low precipitation and strong rainfall seasonality in the previous year, coupled with reduced seasonality in the current year. The risk of infection in humans seems to be also related to the state of vegetation in the previous and the current years. Over the years, the risk of WNV circulation remained consistent in the south-western region with some expansion of the risk to the eastern and northern regions (Figure 1.C). The years 2016 and 2020 stood out as the periods with the highest risk of human exposure. Over 20 years, the risk to humans has increased by 38% (Figure 1.D). Although human cases in Spain have only been reported in 2004, 2010, 2016, and from 2020 onward, it should be noted that the spatiotemporal model allows us to identify locations and time periods with environmental conditions like those in years with reported cases. This enables the mapping of human risk in years without detected cases, which might go undetected, possibly due to the asymptomatic or nonspecific nature of many WNV infections.

**Figure 2:**
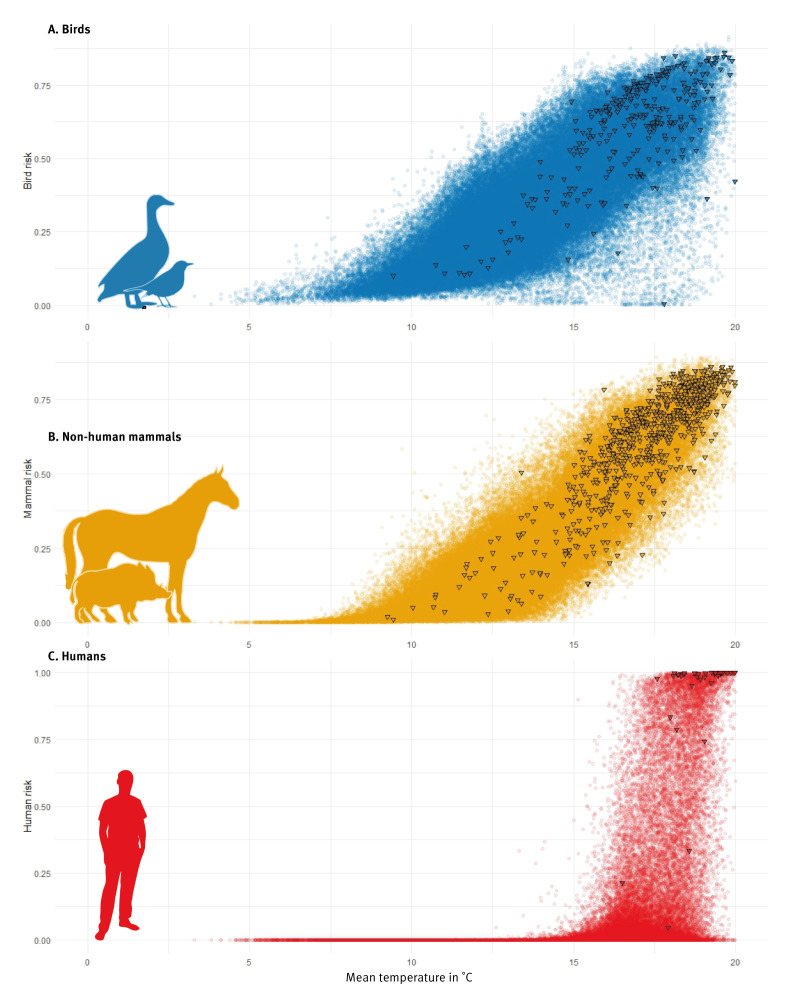
Risk of West Nile virus exposure according to mean temperature, Spain, 2003–2022

In both animals and humans, exposure to the virus was influenced by environmental conditions from both the preceding and current years (Supplementary Table S3). Mean temperature emerges as a recurring variable in areas and years with WNV circulation in birds, non-human mammals, and humans. Viral exposure risk escalates gradually in the following year when mean temperatures exceed 5 °C for birds and 8 °C for non-human mammals, while in humans, the risk of infection increases sharply above 15 °C in the current year (Figure 2). For human infection risk, contemporary mild winters were identified as additional key triggers for outbreaks in the following transmission season across all years. Summarising, areas with high mean annual temperatures exhibit a higher risk of infection, and if these areas experience high temperatures during the winter preceding the transmission season, the risk is further increased. This pattern has been observed across Spain over the 20-year period and at higher administrative units, including autonomous regions and provinces as illustrated in Supplementary Figure S6.

OSTU: Operative Spatio-Temporal Unit.

Each dot represents a specific OSTU (a municipality at a given year from 2003 to 2022) according to its risk. Triangles indicate OSTUs with recorded cases in (A) birds, (B) non-human mammals, and (C) humans. Mean temperature on the X axes are for the previous year in animals (birds and non-human mammals) and for the current year in humans.

### Evaluations of the models and the 1-year lag predictive modelling

All models had sensitivity values ≥ 0.70 (0.93 for human infections) and specificity ≥ 0.65 (0.97 for human infections). The CCR exceeded 0.67 across all models. The AUCs were high, with no model scoring below 0.76. Underprediction rates were low (< 0.118), indicating that they effectively captured most true presences with minimal omission errors. As can be observed in Supplementary Tables S4 and S5, overall, the spatiotemporal models showed slightly better performance when variables from the current year were included, compared with using only variables from the previous year. Sensitivity increased by 0.000–0.016, specificity by 0.005–0.026, and AUC by 0.005–0.015 between predictive-only and full models (Supplementary Table S5). Nevertheless, models based exclusively on previous-year variables still performed well, supporting their potential use in predictive, early-warning frameworks.

### Ensemble models

**Figure 3:**
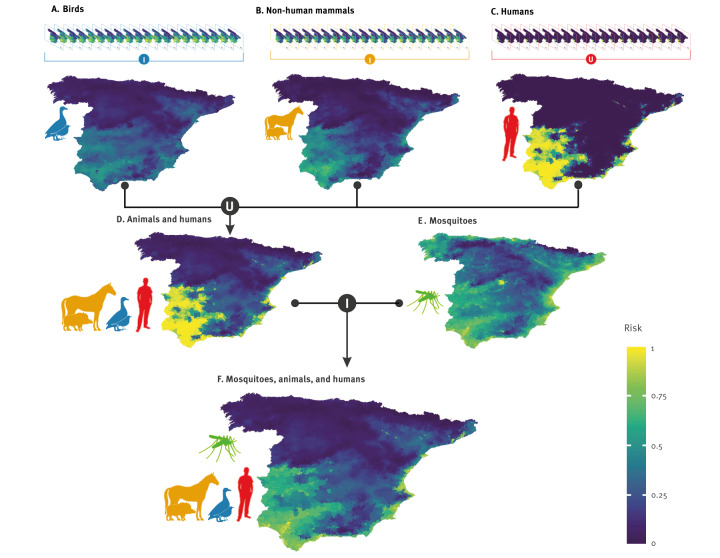
Maps showing variations across Spanish areas of WNV exposure risk^a^ in (A-C) different groups of host species including (A) birds, (B) non-human mammals, and (C) humans, as well as in (D) any host species’ group^b^ and (E) areas favourable for mosquito vectors, as well as (F) areas with both virus circulation risk and suitable conditions for the vectors, Spain, 2003–2022

For birds and non-human mammal components, the intersection of the respective 20 maps provided by the spatiotemporal model provides a single map for each component. These single maps highlight areas with the highest virus circulation intensity and allow to compare birds with non-human mammals in this respect. For birds, the exposure risk is the highest in the south-western quadrant of Spain, and gradually decreases eastwards, with nevertheless high-risk zones on the Mediterranean coast (Figure 3.A). For non-human mammals, high-risk areas are more concentrated in the south-western quadrant than in the case of birds, with lower favourability values elsewhere (Figure 3.B). The fuzzy union of all 20 maps from the human spatiotemporal model yields a single map revealing a strongly polarised risk distribution for people, with few areas of intermediate risk — unlike the more gradual patterns observed in birds and non-human mammals. Very high-risk areas are primarily concentrated in the south-western and eastern coastal regions, with lower-risk areas in between and in the north-east of the country (Figure 3.C). Overall, the exposure risk in birds is more widespread than in other animals and humans.

The fuzzy union of the three risk maps, each representing the spatiotemporal distribution of risk in a single map, identifies areas where there is a risk of virus circulation in at least one of its components: birds, non-human mammals, or humans (Figure 3.D). The intersection of this result with the spatial vector model (Figure 3.E) highlights the southern half of the country, the Mediterranean coastal zone and areas of the Ebro valley as areas where WNV circulation risk in birds, non-human mammals, and humans coincide with highly favourable vector conditions (Figure 3.F).

*I*: fuzzy intersection of the models; *U*: fuzzy union of the models; WNV: West Nile virus.

^a^ Based on spatiotemporal models.

^b^ Based on WNVs circulation in any of the group species (i.e. birds, non-human mammals, and humans) within an area.

Spatiotemporal models using only variables from the previous year, aimed at maximising predictive power, did not differ significantly in terms of risk distribution. In fact, the models for birds, non-human mammals, and humans — along with their combined output — produced remarkably similar maps, as illustrated in Supplementary Figure S7. The variables included in these models did not differ substantially either, as the original explanatory models already contained a high proportion of lagged predictors (75% in birds, 71% in mammals, 40% in humans), highlighting the predictive and explanatory value of environmental conditions from the previous year, as shown in Supplementary Table S6.

## Discussion

Our results reveal an overall increase in WNV circulation risk across Spain between 2003 and 2022, with increases of 19% in birds, 17% in non-human mammals, and 38% in humans. Spatially, the risk is concentrated in the south-western quadrant of the country and along the Mediterranean coastal zone, with occasional expansions towards the northern and eastern regions. Temperature emerged as a key variable triggering exposure risk across all groups. Locations experiencing mean annual temperatures above 5 °C in birds and 8 °C in non-human mammals showed a higher probability of exposure in the following year. In humans, the local risk increased sharply in years with mean temperatures above 15 °C and mild winters. Predictive models based solely on previous-year variables yielded results comparable to contemporaneous models, reinforcing their value for early warning and risk anticipation.

While spatial models identify risk areas based on shared environmental characteristics [53], spatiotemporal models capture variability in the risk across space and time [54]. Human cases show greater interannual fluctuation compared with animal exposure, with only six of the 20 study years reporting human cases. The observed disparity between human case detections and model-predicted high-risk areas suggests that human infections may be more temporally stable than surveillance records indicate. This likely reflects under-reporting due to asymptomatic or mild infections, in addition to variability in surveillance sensitivity, diagnostic awareness, and testing effort across years. Increased awareness of WNV circulation and improved diagnostic capacity in recent years may explain the consistent annual detection of cases since 2020, compared with the sporadic reports in 2004, 2010, and 2016. Spatiotemporal modelling thus provides a robust framework for identifying persistent hotspots masked by sporadic surveillance.

Each component of the WNV transmission cycle showed distinct spatial and temporal patterns of circulation risk, underscoring the limitations of studies focused on a single component [55]. Although viral circulation in natural reservoirs is widespread and stable over time, spillover risk to mammals, and especially to humans remains apparently limited. Greater mosquito exposure in wild and domestic mammals, combined with more intensive active animal surveillance, may explain these differences [8]. Human cases represent just the tip of the iceberg in the broader picture of virus circulation, as they are relatively rare and often detected only after the virus is already widely circulating. Integrating vector, reservoir, and dead-end host models into a single map revealed high-risk areas in the south-west and eastern coast, consistent with previous studies [8,56] and accurately predicted human and animal cases in 2023 and during the major 2024 outbreak [13]. This highlights the strength of a One Health approach that merges multi-sectoral data and expertise from veterinary, medical, and ecological disciplines to establish science-based policies for WNV management.

Temperature variation is a critical driver of WNV exposure dynamics in both animals and humans. Elevated temperatures enhance viral replication, mosquito abundance, and transmission rates [1,57]. Our models reveal consistent 20-year temperature thresholds linked to exposure. Mammals require higher environmental temperatures than birds, while human cases are associated with even warmer conditions and milder winters. This suggests that viral circulation primarily occurs in avian populations (with average temperatures above 5 °C), which may therefore serve as effective sentinels. As average temperatures rise (above 8 °C), increased mosquito abundance may favour spillovers from avian hosts to exposed mammals, given that many vector species are primarily ornithophilic but opportunistic [58]. Warmer winters further reduce mosquito overwintering mortality, boosting spring populations [57]. These expanded vector populations exploit both avian and mammalian hosts, including humans (above an average temperature of 15 °C), escalating spillover risk. Climate change-induced temperature increases may prolong this trend, increasing the risk in endemic areas and spreading to previously unaffected regions [59]. This trend may help explain the gradual increase in exposure risk observed across all WNV components during our study period, potentially influenced by broader climatic changes.

By advancing spatiotemporal models to incorporate only lagged environmental variables, we achieved accurate virus exposure forecasts up to a year in advance without sacrificing explanatory power. This aligns with European surveillance guidelines that promote environmentally informed, proactive interventions [60]. Specifically, these findings inform early targeted vector control (e.g. source reduction, pre-emptive larvicide applications in predicted hotspots), optimised resource allocation (e.g. trap deployment), and tailored risk communication to vulnerable communities before peak transmission.

As with any large-scale modelling framework, our study has limitations that should be noted. Incorporating serological data from human populations would offer a more realistic picture than relying solely on clinical case reports, given the high proportion of asymptomatic infections. Likewise, including demographic, behavioural, and socioeconomic variables (e.g. mobility, and healthcare access) could improve our understanding of vulnerability patterns. Temporal patterns in animal seropositivity must also be interpreted cautiously, as they may reflect past exposure events without precise infection timing. Future models would benefit from the inclusion of infected vector data, and information on the virus lineage, enhancing spatial and temporal resolution. While our models identify favourable conditions during years or in areas without reported cases, these risk estimates should be interpreted conservatively until corroborated by surveillance data.

Implementing context-specific vector control measures against *Culex* mosquitoes is essential to reduce vector densities and interrupt transmission [61]. These measures include eliminating standing water in urban and peri-urban areas (e.g. maintaining drainage systems, repairing leaks, and avoiding water accumulation in containers), managing irrigation canals and rainwater systems, and minimising water storage. Educational initiatives targeting residents and facility managers are also essential, given the adaptability of *Culex* to diverse habitats, including underground deposits and urban catch basins. In agricultural areas, measures emphasise the rational management of irrigation practices to avoid standing water, periodic maintenance of reservoirs and water storage ponds, and the application of biological or chemical larvicides in rice fields or other flood-irrigated crops where *Culex* species commonly breed [62]. Farmers and workers should be engaged in surveillance and habitat management, as their early intervention is critical to prevent large-scale emergence. Risk-based prioritisation also helps optimise resource use, focusing control efforts on high-priority areas to maximise cost-effectiveness and public health impact. This integrated approach not only strengthens cross-sector collaboration, a core component of the One Health concept, but also aligns with international recommendations for addressing vector-borne diseases. 

## Conclusion

Our results reveal an overall increase in WNV circulation risk in animals and humans across Spain between 2003 and 2022. They also suggest that annual human WNV transmission may be more stable than evidenced by surveillance, possibly due to asymptomatic or misdiagnosed cases. Moreover, the approach developed here, by combining different disciplines to improve understanding, anticipate and adapt to climate-driven shifts in WNV dynamics, exemplifies operational One Health. This also facilitates coordinated preventative action across human, veterinary, and environmental health sectors.

## Data Availability

All relevant data are included in the manuscript and its Supplementary material. Additional data supporting the findings of this study are available from the corresponding author upon reasonable request.

## Supplementary Data

839000 Supplementary Material
