# Supplementary material for: Predicting West Nile virus circulation: a 20-year spatiotemporal study in humans and animals in Spain, 2003 to 2022
Source: Euro Surveill. 2026 Apr 23;31(16):2500535. doi: 10.2807/1560-7917.ES.2026.31.16.2500535 (PMC13109697; doi:10.2807/1560-7917.ES.2026.31.16.2500535)
Supplement: Supplementary Material [file 25-00535_SupplementaryMaterial.pdf]

Supplementary Material

This supplementary material is hosted by Eurosurveillance as supporting information alongside the article "Predicting West Nile Virus Circulation: A 20-Year (2003-2022) Spatiotemporal Study in Humans and Animals in Spain", on behalf of the authors, who remain responsible for the accuracy and appropriateness of the content. The same standards for ethics, copyright, attributions and permissions as for the article apply. Supplements are not edited by *Eurosurveillance* and the journal is not responsible for the maintenance of any links or email addresses provided therein.

Supplementary Figure S1. Spatial distribution of Culex mosquito vectors in Spain. Orange municipalities indicate locations where mosquitoes were detected.

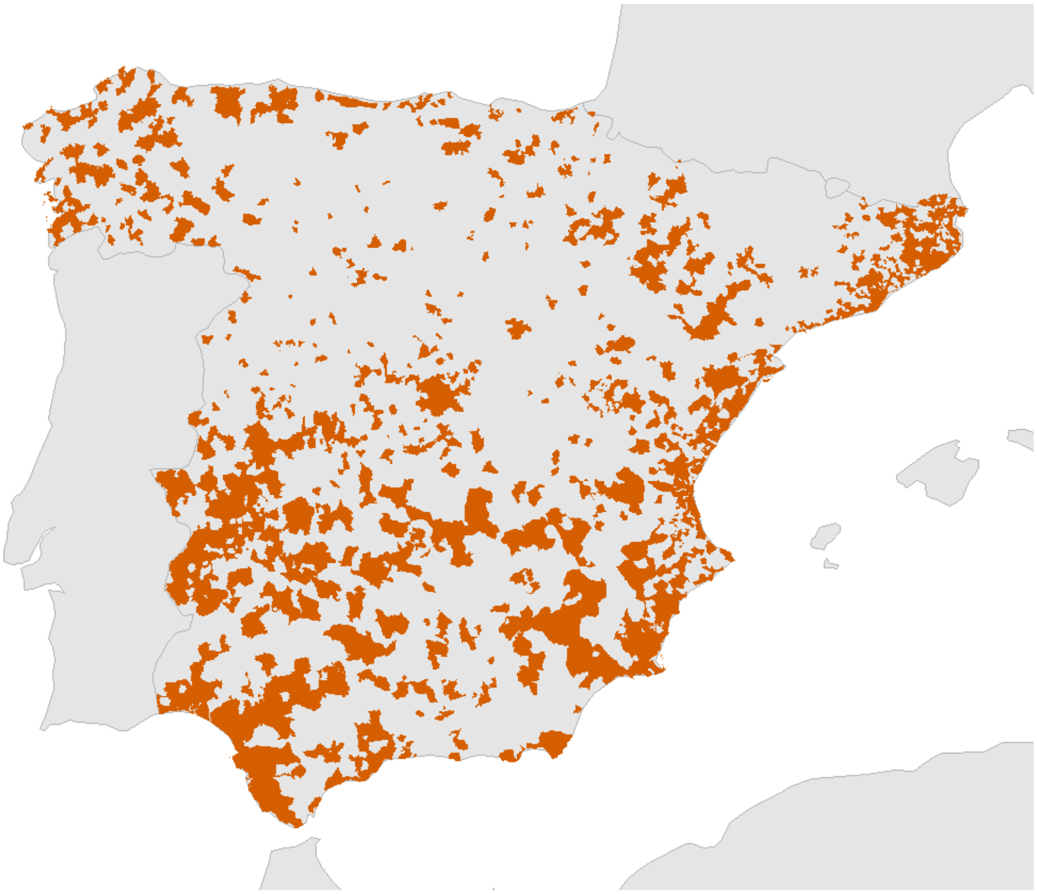

Supplementary Figure S2. Spatial distribution of surveyed municipalities with positive (orange) and negative (blue) West Nile virus exposure detected in birds over the years.

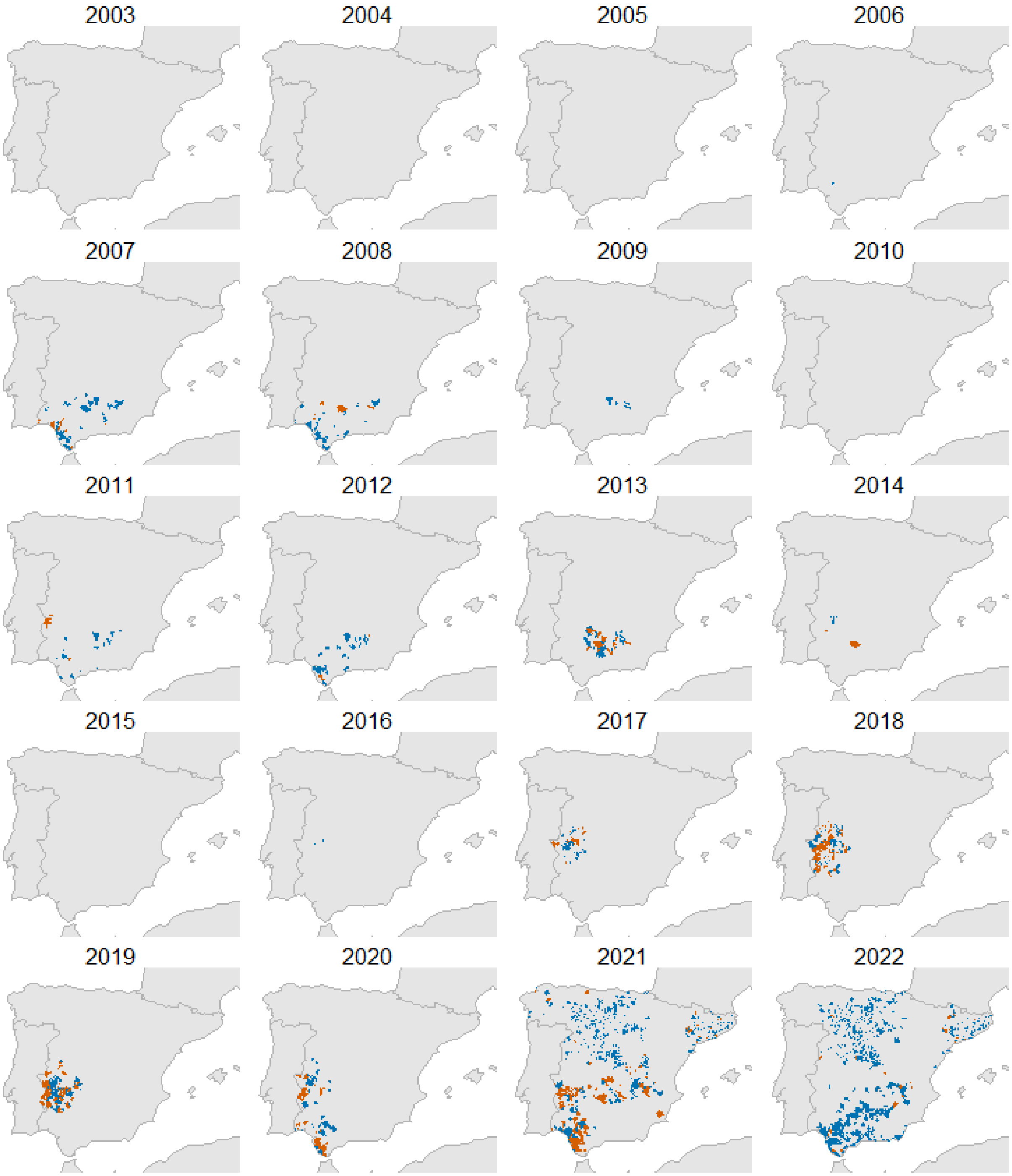

Supplementary Figure S3. Spatial distribution of surveyed municipalities with positive (orange) and negative (blue) West Nile virus exposure detected in non-human mammals over the years.

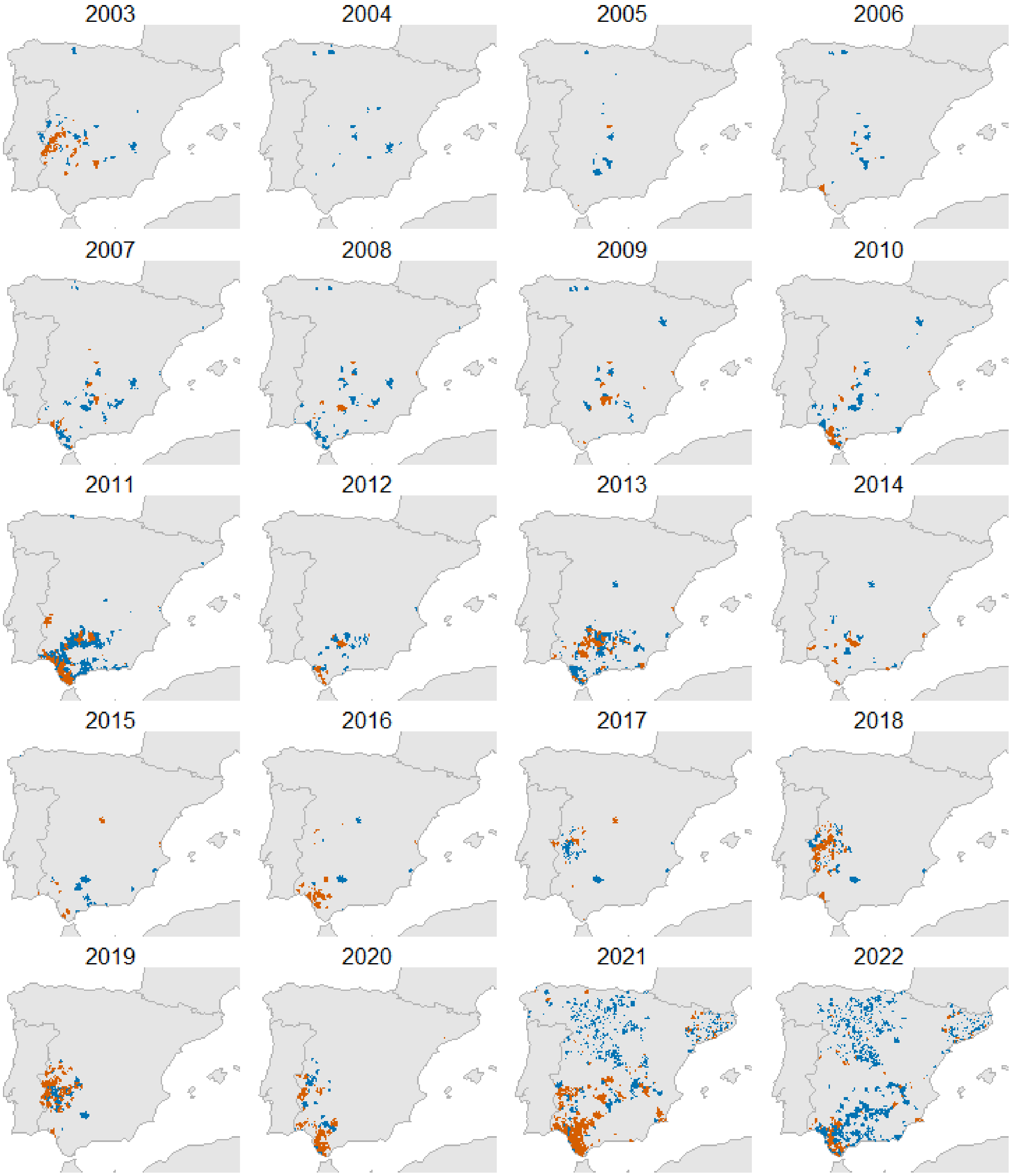

Supplementary Figure S4. Spatial distribution of municipalities with human West Nile virus cases (orange) over the years.

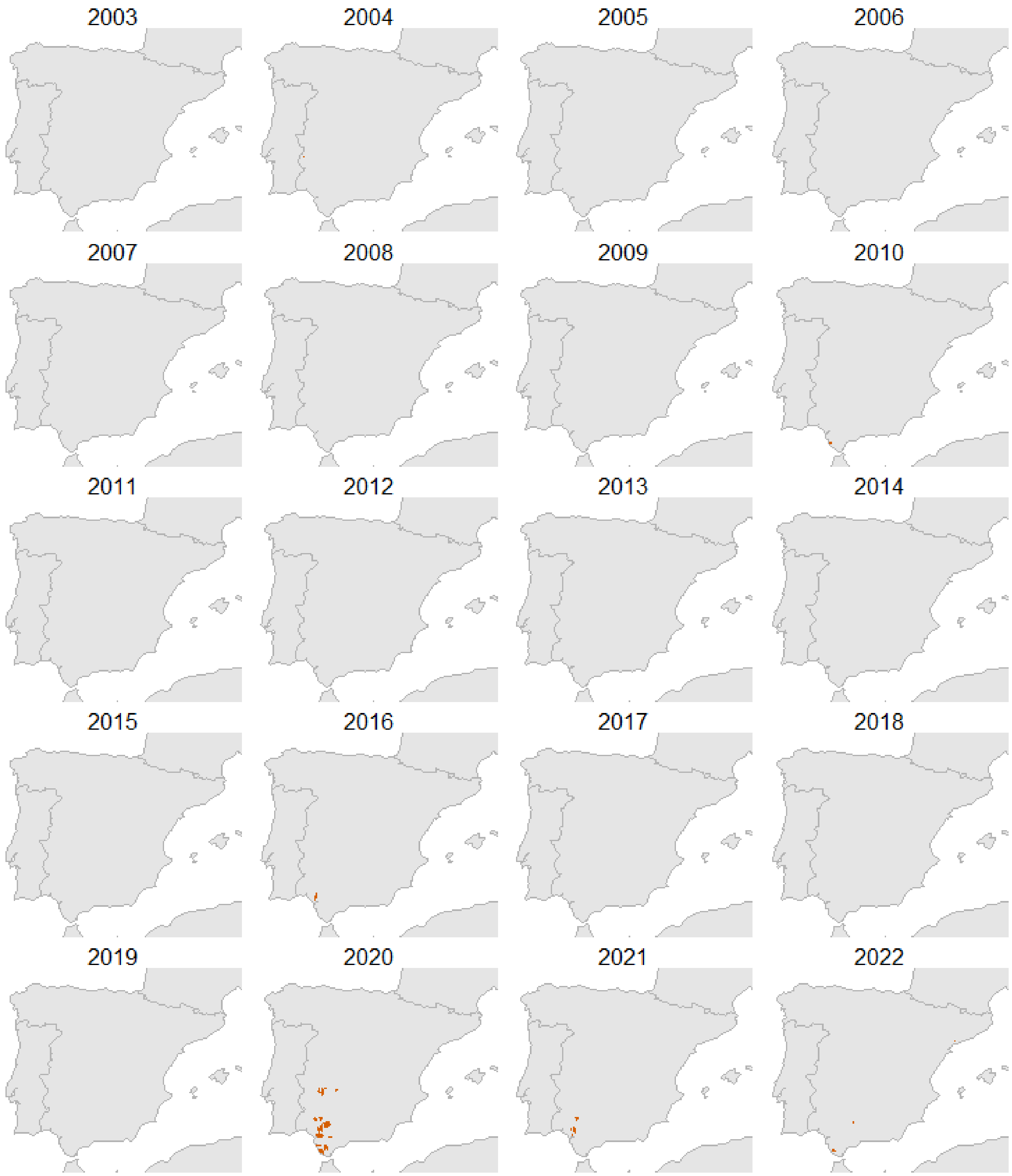

Supplementary Table S1. Class, order, family and species of the different birds and non-human mammals analyzed for West Nile virus exposure.

**Class**	**Order**	**Family**	**Species**
Aves	Accipitriformes	Accipitridae	*Accipiter gentilis*
Aves	Accipitriformes	Accipitridae	*Accipiter nisus*
Aves	Accipitriformes	Accipitridae	*Aegypius monachus*
Aves	Accipitriformes	Accipitridae	*Aquila adalberti*
Aves	Accipitriformes	Accipitridae	*Aquila chrysaetos*
Aves	Accipitriformes	Accipitridae	*Aquila fasciata*
Aves	Accipitriformes	Accipitridae	*Buteo buteo*
Aves	Accipitriformes	Accipitridae	*Circaetus gallicus*
Aves	Accipitriformes	Accipitridae	*Circus aeruginosus*
Aves	Accipitriformes	Accipitridae	*Circus pygargus*
Aves	Accipitriformes	Accipitridae	*Elanus caeruleus*
Aves	Accipitriformes	Accipitridae	*Gypaetus barbatus*
Aves	Accipitriformes	Accipitridae	*Gyps fulvus*
Aves	Accipitriformes	Accipitridae	*Hieraaetus pennatus*
Aves	Accipitriformes	Accipitridae	*Milvus migrans*
Aves	Accipitriformes	Accipitridae	*Milvus milvus*
Aves	Accipitriformes	Accipitridae	*Neophron percnopterus*
Aves	Accipitriformes	Pandionidae	*Pandion haliaetus*
Aves	Accipitriformes	Accipitridae	*Parabuteo unicinctus*
Aves	Accipitriformes	Accipitridae	*Pernis apivorus*
Aves	Anseriformes	Anatidae	*Anas platyrhynchos*
Aves	Anseriformes	Anatidae	*Anser indicus*
Aves	Anseriformes	Anatidae	*Cairina moschata*
Aves	Anseriformes	Anatidae	*Chen canagica*
Aves	Anseriformes	Anatidae	*Dendrocygna autumnalis*
Aves	Anseriformes	Anatidae	*Netta peposaca*
Aves	Apodiformes	Apodidae	*Apus apus*
Aves	Apodiformes	Apodidae	*Apus pallidus*
Aves	Caprimulgiformes	Caprimulgidae	*Caprimulgus ruficollis*

Supplementary Table S1 (continued). Class, order, family and species of the different birds and non-human mammals analyzed for West Nile virus exposure.

Class	Order	Family	Species
Aves	Casuariiformes	Casuariidae	*Dromaius novaehollandiae*
Aves	Charadriiformes	Burhinidae	*Burhinus oedicnemus*
Aves	Charadriiformes	Scolopacidae	*Calidris alpina*
Aves	Charadriiformes	Laridae	*Chroicocephalus ridibundus*
Aves	Charadriiformes	Laridae	*Ichthyaetus audouinii*
Aves	Charadriiformes	Laridae	*Larus fuscus*
Aves	Charadriiformes	Laridae	*Larus michahellis*
Aves	Charadriiformes	Stercorariidae	*Stercorarius skua*
Aves	Ciconiiformes	Ciconiidae	*Ciconia ciconia*
Aves	Ciconiiformes	Ciconiidae	*Ciconia nigra*
Aves	Columbiformes	Columbidae	*Columba livia*
Aves	Columbiformes	Columbidae	*Streptopelia decaocto*
Aves	Coraciiformes	Alcedinidae	*Alcedo atthis*
Aves	Coraciiformes	Meropidae	*Merops apiaster*
Aves	Coraciiformes	Upupidae	*Upupa epops*
Aves	Cuculiformes	Cuculidae	*Cuculus canorus*
Aves	Falconiformes	Falconidae	*Falco columbarius*
Aves	Falconiformes	Falconidae	*Falco naumanni*
Aves	Falconiformes	Falconidae	*Falco peregrinus*
Aves	Falconiformes	Falconidae	*Falco tinnunculus*
Aves	Galliformes	Phasianidae	*Pavo cristatus*
Aves	Gruiformes	Rallidae	*Fulica atra*
Aves	Gruiformes	Rallidae	*Gallinula chloropus*
Aves	Gruiformes	Gruidae	*Grus grus*
Aves	Otidiformes	Otididae	*Otis tarda*
Aves	Passeriformes	Acrocephalidae	*Acrocephalus scirpaceus*
Aves	Passeriformes	Aegithalidae	*Aegithalos caudatus*
Aves	Passeriformes	Estrildidae	*Amandava amandava*
Aves	Passeriformes	Motacillidae	*Anthus pratensis*

Supplementary Table S1 (continued). Class, order, family and species of the different birds and non-human mammals analyzed for West Nile virus exposure.

Class	Order	Family	Species
Aves	Passeriformes	Fringillidae	*Carduelis carduelis*
Aves	Passeriformes	Cettiidae	*Cettia cetti*
Aves	Passeriformes	Fringillidae	*Chloris chloris*
Aves	Passeriformes	Cisticolidae	*Cisticola juncidis*
Aves	Passeriformes	Corvidae	*Coloeus monedula*
Aves	Passeriformes	Corvidae	*Corvus corax*
Aves	Passeriformes	Paridae	*Cyanistes caeruleus*
Aves	Passeriformes	Corvidae	*Cyanopica cooki*
Aves	Passeriformes	Corvidae	*Cyanopica cyanus*
Aves	Passeriformes	Hirundinidae	*Delichon urbicum*
Aves	Passeriformes	Muscicapidae	*Erithacus rubecula*
Aves	Passeriformes	Estrildidae	*Estrilda astrild*
Aves	Passeriformes	Euplectidae	*Euplectes afer*
Aves	Passeriformes	Muscicapidae	*Ficedula hypoleuca*
Aves	Passeriformes	Fringillidae	*Fringilla coelebs*
Aves	Passeriformes	Alaudidae	*Galerida cristata*
Aves	Passeriformes	Corvidae	*Garrulus glandarius*
Aves	Passeriformes	Acrocephalidae	*Hippolais polyglotta*
Aves	Passeriformes	Hirundinidae	*Hirundo rustica*
Aves	Passeriformes	Acrocephalidae	*Lanius senator*
Aves	Passeriformes	Muscicapidae	*Luscinia megarhynchos*
Aves	Passeriformes	Muscicapidae	*Luscinia svecica*
Aves	Passeriformes	Emberizidae	*Miliaria calandra*
Aves	Passeriformes	Motacillidae	*Motacilla alba*
Aves	Passeriformes	Oriolidae	*Oriolus oriolus*
Aves	Passeriformes	Paridae	*Parus major*
Aves	Passeriformes	Passeridae	*Passer domesticus*
Aves	Passeriformes	Passeridae	*Passer hispaniolensis*
Aves	Passeriformes	Passeridae	*Passer montanus*

Supplementary Table S1 (continued). Class, order, family and species of the different birds and non-human mammals analyzed for West Nile virus exposure.

Class	Order	Family	Species
Aves	Passeriformes	Passeridae	*Petronia petronia*
Aves	Passeriformes	Phylloscopidae	*Phylloscopus collybita*
Aves	Passeriformes	Phylloscopidae	*Phylloscopus trochilus*
Aves	Passeriformes	Corvidae	*Pica pica*
Aves	Passeriformes	Corvidae	*Pyrrhocorax pyrrhocorax*
Aves	Passeriformes	Hirundinidae	*Riparia riparia*
Aves	Passeriformes	Muscicapidae	*Saxicola torquatus*
Aves	Passeriformes	Fringillidae	*Serinus serinus*
Aves	Passeriformes	Sturnidae	*Sturnus unicolor*
Aves	Passeriformes	Sturnidae	*Sturnus vulgaris*
Aves	Passeriformes	Sylviidae	*Sylvia atricapilla*
Aves	Passeriformes	Sylviidae	*Sylvia melanocephala*
Aves	Passeriformes	Troglodytidae	*Troglodytes troglodytes*
Aves	Passeriformes	Turdidae	*Turdus merula*
Aves	Passeriformes	Turdidae	*Turdus philomelos*
Aves	Pelecaniformes	Ardeidae	*Ardea cinerea*
Aves	Pelecaniformes	Ardeidae	*Ardea purpurea*
Aves	Pelecaniformes	Ardeidae	*Bubulcus ibis*
Aves	Pelecaniformes	Phalacrocoracidae	*Phalacrocorax*
Aves	Pelecaniformes	Threskiornithidae	*Platalea leucorodia*
Aves	Pelecaniformes	Threskiornithidae	*Plegadis falcinellus*
Aves	Pelecaniformes	Threskiornithidae	*Threskiornis aethiopicus*
Aves	Piciformes	Picidae	*Dendrocopos major*
Aves	Piciformes	Picidae	*Jynx torquilla*
Aves	Rheiformes	Rheidae	*Rhea americana*
Aves	Strigiformes	Strigidae	*Asio otus*
Aves	Strigiformes	Strigidae	*Athene noctua*
Aves	Strigiformes	Strigidae	*Bubo bubo*
Aves	Strigiformes	Strigidae	*Otus scops*

Supplementary Table S1 (continued). Class, order, family and species of the different birds and non-human mammals analyzed for West Nile virus exposure.

**Class**	**Order**	**Family**	**Species**
Aves	Strigiformes	Strigidae	*Strix aluco*
Aves	Strigiformes	Strigidae	*Tyto alba*
Aves	Struthioniformes	Struthionidae	*Struthio camelus*
Aves	Suliformes	Sulidae	*Morus bassanus*
Mammalia	Artiodactyla	Bovidae	*Aepyceros melampus*
Mammalia	Artiodactyla	Cervidae	*Alces alces*
Mammalia	Artiodactyla	Bovidae	*Ammotragus lervia*
Mammalia	Artiodactyla	Suidae	*Babyrousa babyrussa*
Mammalia	Artiodactyla	Bovidae	*Bison bonasus*
Mammalia	Artiodactyla	Bovidae	*Bos javanicus*
Mammalia	Artiodactyla	Bovidae	*Bos mutus*
Mammalia	Artiodactyla	Bovidae	*Bos taurus indicus*
Mammalia	Artiodactyla	Bovidae	*Boselaphus tragocamelus*
Mammalia	Artiodactyla	Bovidae	*Budorcas taxicolor*
Mammalia	Artiodactyla	Camelidae	*Camelus dromedarius*
Mammalia	Artiodactyla	Bovidae	*Capra aegagrus hircus*
Mammalia	Artiodactyla	Bovidae	*Capra pyrenaica*
Mammalia	Artiodactyla	Cervidae	*Cervus elaphus*
Mammalia	Artiodactyla	Bovidae	*Connochaetes gnou*
Mammalia	Artiodactyla	Cervidae	*Dama dama*
Mammalia	Artiodactyla	Bovidae	*Damaliscus pygargus phillipsi*
Mammalia	Artiodactyla	Monodontidae	*Delphinapterus leucas*
Mammalia	Artiodactyla	Tayassuidae	*Dicotyles tajacu*
Mammalia	Artiodactyla	Bovidae	*Duiker azul*
Mammalia	Artiodactyla	Bovidae	*Eudorcas thomsonii*
Mammalia	Artiodactyla	Bovidae	*Gazella dorcas*
Mammalia	Artiodactyla	Giraffidae	*Giraffa camelopardalis rothschildi*
Mammalia	Artiodactyla	Camelidae	*Lama glama*
Mammalia	Artiodactyla	Cervidae	*Muntiacus reevesi*

Supplementary Table S1 (continued). Class, order, family and species of the different birds and non-human mammals analyzed for West Nile virus exposure.

Class	Order	Family	Species
Mammalia	Artiodactyla	Bovidae	*Nanger dama*
Mammalia	Artiodactyla	Bovidae	*Oryx dammah*
Mammalia	Artiodactyla	Bovidae	*Ovis orientalis aries*
Mammalia	Artiodactyla	Bovidae	*Ovis orientalis musimon*
Mammalia	Artiodactyla	Suidae	*Potamochoerus porcus*
Mammalia	Artiodactyla	Cervidae	*Rusa alfredi*
Mammalia	Artiodactyla	Suidae	*Sus scrofa*
Mammalia	Artiodactyla	Bovidae	*Syncerus caffer nanus*
Mammalia	Artiodactyla	Bovidae	*Taurotragus oryx*
Mammalia	Artiodactyla	Bovidae	*Tragelaphus eurycerus*
Mammalia	Artiodactyla	Bovidae	*Tragelaphus spekii*
Mammalia	Artiodactyla	Delphinidae	*Tursiops truncatus*
Mammalia	Carnivora	Ursidae	*Ailuropoda melanoleuca*
Mammalia	Carnivora	Ailuridae	*Ailurus fulgens*
Mammalia	Carnivora	Mustelidae	*Aonyx cinereus*
Mammalia	Carnivora	Viverridae	*Arctictis binturong*
Mammalia	Carnivora	Canidae	*Canis familiaris*
Mammalia	Carnivora	Canidae	*Canis lupus signatus*
Mammalia	Carnivora	Eupleridae	*Cryptoprocta ferox*
Mammalia	Carnivora	Viverridae	*Genetta genetta*
Mammalia	Carnivora	Ursidae	*Helarctos malayanus*
Mammalia	Carnivora	Herpestidae	*Helogale parvula*
Mammalia	Carnivora	Hyaenidae	*Hyaenidae*
Mammalia	Carnivora	Felidae	*Leptailurus serval*
Mammalia	Carnivora	Mustelidae	*Lutra lutra*
Mammalia	Carnivora	Felidae	*Lynx lynx*
Mammalia	Carnivora	Mephitidae	*Mephitidae*
Mammalia	Carnivora	Mustelidae	*Mustela lutreola*
Mammalia	Carnivora	Procyonidae	*Nasua*

Supplementary Table S1 (continued). Class, order, family and species of the different birds and non-human mammals analyzed for West Nile virus exposure.

Class	Order	Family	Species
Mammalia	Carnivora	Odobenidae	*Odobenus rosmarus*
Mammalia	Carnivora	Otariidae	*Otaria flavescens*
Mammalia	Carnivora	Felidae	*Panthera leo*
Mammalia	Carnivora	Felidae	*Panthera leo bleyenberghi*
Mammalia	Carnivora	Felidae	*Panthera leo leo*
Mammalia	Carnivora	Felidae	*Panthera onca*
Mammalia	Carnivora	Felidae	*Panthera pardus*
Mammalia	Carnivora	Felidae	*Panthera pardus kotiya*
Mammalia	Carnivora	Felidae	*Panthera tigris sumatrae*
Mammalia	Carnivora	Felidae	*Panthera tigris tigris*
Mammalia	Carnivora	Phocidae	*Phoca vitulina*
Mammalia	Carnivora	Procyonidae	*Potos flavus*
Mammalia	Carnivora	Procyonidae	*Procyon lotor*
Mammalia	Carnivora	Herpestidae	*Suricata suricatta*
Mammalia	Carnivora	Ursidae	*Ursus arctos*
Mammalia	Carnivora	Ursidae	*Ursus thibetanus*
Mammalia	Cingulata	Dasypodidae	*Dasypodidae*
Mammalia	Diprotodontia	Macropodidae	*Macropus rufogriseus*
Mammalia	Perissodactyla	Rhinocerotidae	*Ceratotherium simum*
Mammalia	Perissodactyla	Equidae	*Equus asinus*
Mammalia	Perissodactyla	Equidae	*Equus asinus x Equus caballus*
Mammalia	Perissodactyla	Equidae	*Equus caballus*
Mammalia	Perissodactyla	Equidae	*Equus ferus caballus*
Mammalia	Perissodactyla	Equidae	*Equus quagga boehmi*
Mammalia	Perissodactyla	Equidae	*Equus zebra*
Mammalia	Perissodactyla	Tapiridae	*Tapirus indicus*
Mammalia	Primates	Callitrichidae	*Callithrix penicillata*
Mammalia	Primates	Cercopithecidae	*Cercocebus atys*
Mammalia	Primates	Cercopithecidae	*Cercopithecus hamlyni*

Supplementary Table S1 (continued). Class, order, family and species of the different birds and non-human mammals analyzed for West Nile virus exposure.

Class	Order	Family	Species
Mammalia	Primates	Cercopithecidae	*Cercopithecus mona*
Mammalia	Primates	Cercopithecidae	*Cercopithecus neglectus*
Mammalia	Primates	Cercopithecidae	*Colobus*
Mammalia	Primates	Cercopithecidae	*Erythrocebus patas*
Mammalia	Primates	Lemuridae	*Eulemur albifrons*
Mammalia	Primates	Lemuridae	*Eulemur macaco*
Mammalia	Primates	Lemuridae	*Eulemur mongoz*
Mammalia	Primates	Hominidae	*Gorilla gorilla gorilla*
Mammalia	Primates	Hylobatidae	*Hylobates lar*
Mammalia	Primates	Hylobatidae	*Hylobates muelleri*
Mammalia	Primates	Lemuridae	*Lemur catta*
Mammalia	Primates	Lemuridae	*Lemur rubriventer*
Mammalia	Primates	Callitrichidae	*Leontopithecus chrysomelas*
Mammalia	Primates	Cercopithecidae	*Lophocebus aterrimus*
Mammalia	Primates	Cercopithecidae	*Macaca fuscata*
Mammalia	Primates	Cercopithecidae	*Macaca sylvanus*
Mammalia	Primates	Cercopithecidae	*Mandrillus leucophaeus*
Mammalia	Primates	Cercopithecidae	*Mandrillus sphinx*
Mammalia	Primates	Cercopithecidae	*Miopithecus ogouensis*
Mammalia	Primates	Hylobatidae	*Nomascus gabriellae*
Mammalia	Primates	Lorisidae	*Nycticebus pygmaeus*
Mammalia	Primates	Hominidae	*Pan troglodytes*
Mammalia	Primates	Cercopithecidae	*Papio*
Mammalia	Primates	Hominidae	*Pongo pygmaeus*
Mammalia	Primates	Indriidae	*Varecia rubra*
Mammalia	Primates	Indriidae	*Varecia variegata*
Mammalia	Proboscidea	Elephantidae	*Elephas maximus*
Mammalia	Proboscidea	Elephantidae	*Loxodonta africana*
Mammalia	Rodentia	Caviidae	*Dolichotis patagonum*
Mammalia	Rodentia	Caviidae	*Hydrochoerus hydrochaeris*
Mammalia	Rodentia	Hystricidae	*Hystrix africaeaustralis*
Mammalia	Rodentia	Hystricidae	*Hystrix cristata*

Supplementary Table S2. Explanatory variables used in the West Nile virus spatial and spatiotemporal models.

Factor	Variable Abbreviation	Description	Units	Source
Anthropic	Dens_pop	Population density	people/km²	(1)
Topo-hydrographic	Altitude	Altitude	meters	(2)
	Slope	Slope	degrees	(3)
	Distance to water source	Distance to rivers and water bodies	meters	(4)
	water-wetness_unique	Water availability, including permanent water, temporary water, permanent wetness, or temporary wetness	%	(5)
	water-wetness_q	Permanent water, temporary water, permanent wet and temporary wet. Quantitavity, higher water available higher values	%	
	water	Water (temporal or permanent)	%	
	wetness	Wetness (temporal or permanent)	%	
Ecosystem	NDVI mean	Mean value of the NDVI throughout the year	unitless	(6)
	NDVI min	Minimum value of NDVI throughout the year	unitless	
	NDVI max	Maximum value of NDVI throughout the year	unitless	
	NDVI difference	Range between the maximum and minimum value of NDVI throughout the year	unitless	
Weather	T mean	Mean temperature throughout the year	°C	(7)
	T min	Minimum temperature throughout the year	°C	
	T max	Maximum temperature throughout the year	°C	
	T range	Difference between the maximum and minimum temperatures throughout the year	°C	
	P	Accumulated precipitation throughout the year	millimeters	
	P min	Minimum precipitation of the driest month throughout the year	millimeters	
	P max	Maximum precipitation of the wettest month throughout the year	millimeters	
	P seasonality	Precipitation coefficient (standard deviation x 100)/mean) throughout the year	unitless	

(1) Landscan 2023 Global Population Database (https://landscan.ornl.gov)

(2) GTOPO30 (US Geological Survey 1996)
(3) Elaborated from DEM (Digital Elevation Model) using the altitude variable (GTOPO30; US Geological Survey 1996), using the Geographic Information Sistem ArcGIS Desktop 10.3.
(4) Datos Espaciales de Referencia de Andalucia (DERA) (https://www.juntadeandalucia.es/institutodeestadisticaycartografia/dega/datos-espaciales-de-referencia-de-andalucia-dera/descarga-de-informacion)
(5) Copernicus Land Monitoring Service (https://land.copernicus.eu/en/products/high-resolution-layer-water-and-wetness/water-and-wetness-status-2018)
(6) MOD13Q1 v061. LP DAAC - Land Processes Distributed Active Archive Center (https://lpdaac.usgs.gov/products/mod13q1v061/)
(7) Agencia Estatal de Meteorologia (https://www.aemet.es)

Supplementary Equation S1. Favorability Function.

$$F = \frac{\frac{P}{(1-P)}}{\frac{n_1}{n_0} + \frac{P}{(1-P)}}$$

Where F is the favorability value, ranging from 0 to 1. Reflects how favorable local conditions are for the occurrence of the event, in this case, the risk of exposure to the virus. P is the probability of occurrence obtained from logistic regression for a given OGU or OSTU. n_1 and n_0 are the number of presences and absences (OGUs or OSTUs) of the event, respectively, in the study area.

Supplementary Equation S2. Fuzzy Intersection and Fuzzy Union equations.

$$Fuzzy\ Intersection\ (F_{2003} - F_{2022}) = F_{2003} \cap F_{2004} \cap \ldots \cap F_{2022}$$
$$= \min\,(F_{2003}, F_{2004}, \ldots, F_{2022})$$

$$Fuzzy\ Union\ (F_{2003} - F_{2022}) = F_{2003} \cup F_{2004} \cup \ldots \cup F_{2022}$$
$$= \max\,(F_{2003}, F_{2004}, \ldots, F_{2022})$$

Fuzzy Intersection calculates the minimum favorability across all years (2003 to 2022) for each Operative Spatio-temporal Unit (OSTU). Conceptually, this operation identifies areas that were consistently favorable every year. Only locations where all yearly favorability values are high will show high intersection values. Conversely, Fuzzy Union calculates the maximum favorability across all years for each spatial unit. Conceptually, it highlights areas that were favorable in at least one year, representing the cumulative potential risk across the entire period.

Supplementary Table S3. Predictor variables included in the spatial mosquito model, as well as in the spatiotemporal models for West Nile virus in birds, mammals, and humans in Spain during the period 2003–2022. *Estimate* is the value of the coefficient that multiplies the variable value in the logit of the multivariate logistic regression. The *Wald* parameter quantifies the relevance of every variable in the model. (0) Indicates spatiotemporal variables that explain virus detection in the year it is detected, while (-1) refers to spatiotemporal variables from the previous year that explain virus detection in the year it is detected.

	Mosquito		Bird		Non-human mammal		Human	
**Variable**	**Estimate**	**Wald**	**Estimate**	**Wald**	**Estimate**	**Wald**	**Estimate**	**Wald**
Anthropic								
dens_pop	1.04E-03	1.20E+02	-6.77E-04	1.04E+01	-	-	-	-
Topo-hydrographic								
altitude	-1.31E-03	1.10E+02	-	-	-	-	-	-
slope	-	-	-	-	-1.75E-01	1.59E+01	-	-
water-wetness_unique	-	-	-	-	-	-	-	-
water-wetness_q	1.54E+00	3.84E+01					-	-
Ecosystem								
NDVI mean (0)			-	-	-	-	-	-
NDVI mean (-1)			-	-	-	-	1.35E-03	1.97E+01
NDVI min	4.03E-04	8.21E+01						
NDVI min (0)			-	-	4.73E-04	4.93E+01	-	-
NDVI difference (0)			-	-	-	-	1.42E-03	4.17E+01
NDVI difference (-1)			-	-	2.47E-04	2.02E+01	-	-
Weather								
T.mean (0)			-	-	-	-	1.10E+00	9.15E+00
T.mean (-1)			4.24E-01	2.46E+01	3.79E-01	1.29E+02	-	-
T.min (0)			-	-	-	-	5.80E-01	9.71E+00
T.max (0)			-2.20E-01	2.45E+01	-	-	7.29E-01	3.19E+01
T.max (-1)			1.28E-01	2.72E+00	-	-	-2.91E-01	5.58E+00
T.range (-1)			1.22E-01	4.46E+00	-	-	-	-
P (0)			-	-	-	-	-4.01E-03	8.28E+00
P (-1)			-	-	-4.97E-04	6.07E+00	-	-
P max	-1.21E-03	3.95E+00						
P min (0)			-	-	-	-	-	-
P min (-1)			-	-	-4.19E-02	6.58E+00	-3.65E+00	6.43E+00
P seasonality	2.10E-02	9.31E+01						
P seasonality (0)			-	-	-5.00E-03	4.36E+00	-4.75E-02	1.46E+01
P seasonality (-1)			-	-	6.72E-03	6.88E+00	2.83E-02	1.90E+01
Zoogeographic								
mosquito			-	-	-	-	7.22E+00	1.35E+01
Intercept	-4.19E+00	2.05E+02	-8.90E+00	8.66E+01	-8.85E+00	2.05E+02	-5.52E+01	9.29E+01

Supplementary Figure S5. Spatial model for West Nile virus vector distribution.

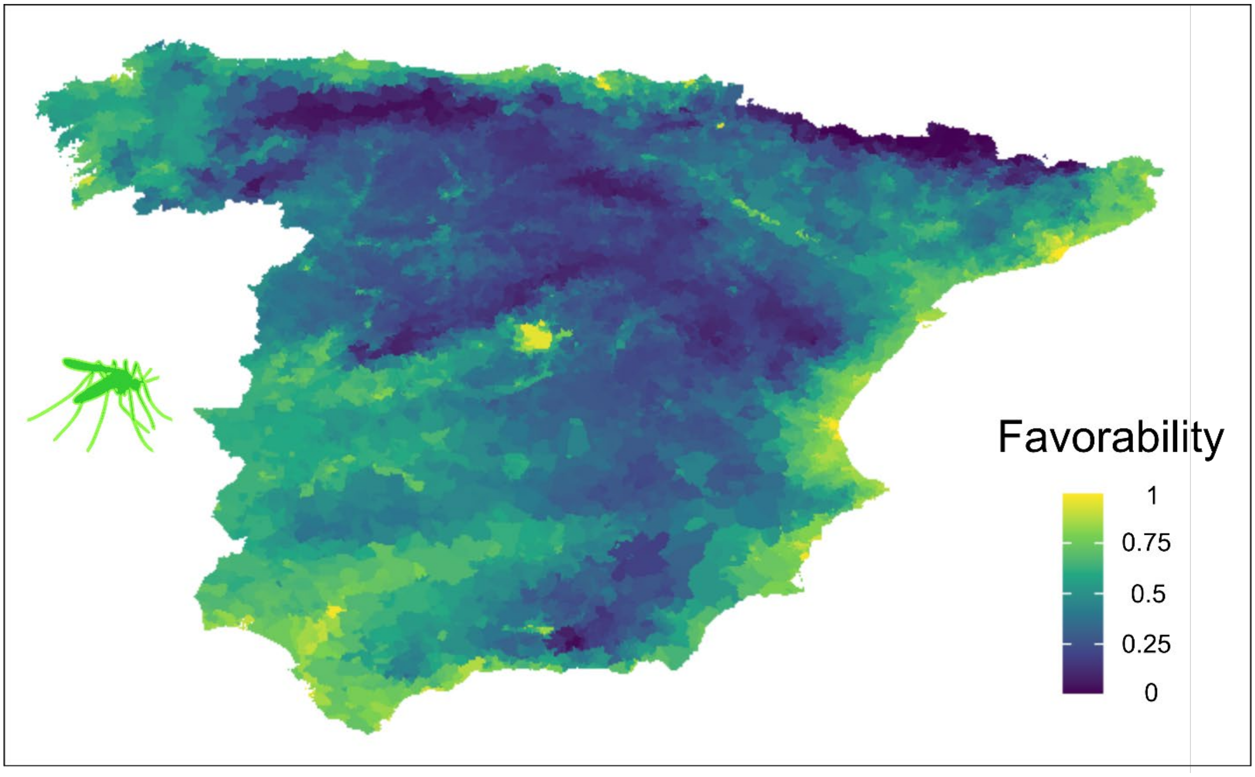

Supplementary Figure S6. Progression of West Nile virus circulation risk in humans according to the minimum temperatures during the winter preceding the transmission season. Effect tested at (A) the national level and higher resolutions, such as (B) autonomous communities (Andalusia) and (C) provinces (Seville). Gray bars indicate the risk, while the red line represents winter temperature.

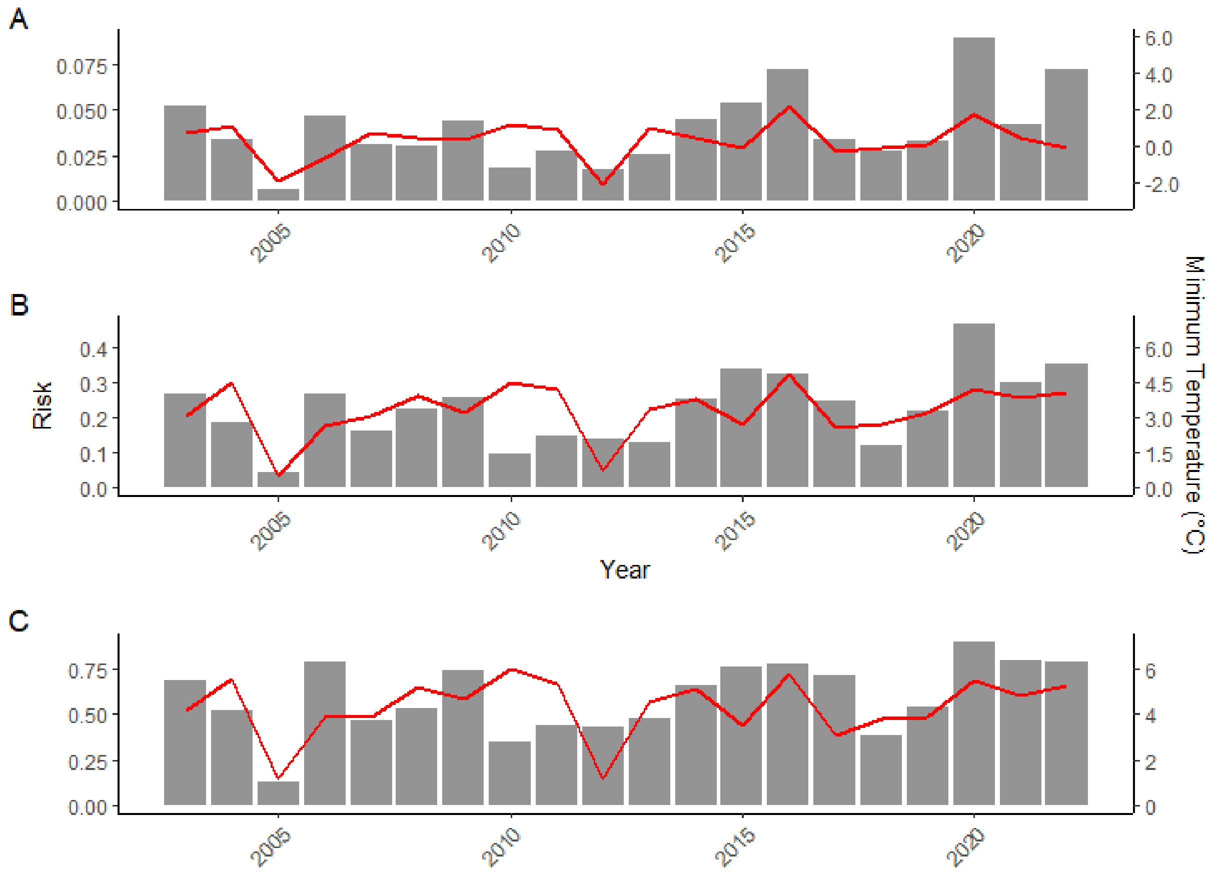

Supplementary Table S4. Comparative assessment of the classification and discrimination capacities of the different spatial (mosquito) and spatiotemporal models (bird, non-human mammal and human). CCR: Correct Classification Rate; TSS: True Skill Statistic; AUC: Area Under the Receiver Operating Characteristic Curve.

	Mosquito	Bird	Non-human mammal	Human
**Sensitivity**	0.696	0.775	0.769	0.932
**Specificity**	0.709	0.647	0.648	0.965
**Under-prediction**	0.065	0.070	0.118	0.000
**Over-prediction**	0.720	0.678	0.551	0.993
**CCR**	0.707	0.670	0.681	0.965
**TSS**	0.405	0.422	0.417	0.896
**AUC**	0.768	0.761	0.767	0.993

Supplementary Table S5. Comparative assessment of the classification and discrimination capacities of the different spatial (mosquito) and spatiotemporal models (bird, non-human mammal and human) considering only spatiotemporal variables from the previous year. CCR: Correct Classification Rate; TSS: True Skill Statistic; AUC: Area Under the Receiver Operating Characteristic Curve.

	Bird	Non-human mammal	Human
**Sensitivity**	0.761	0.753	0.932
**Specificity**	0.642	0.636	0.939
**Under-prediction**	0.074	0.127	0.000
**Over-prediction**	0.685	0.564	0.996
**CCR**	0.663	0.668	0.939
**TSS**	0.403	0.389	0.871
**AUC**	0.754	0.752	0.988

Model performance improved slightly for birds, non-human mammals, and humans when using current-year (and previous year) variables compared to only previous-year variables. The changes in key metrics were as follows: Sensitivity (0.014, 0.016, 0), Specificity (0.005, 0.012, 0.026), Under-prediction (–0.004, –0.009, 0), Over-prediction (–0.007, –0.013, –0.003), CCR (0.007, 0.013, 0.026), TSS (0.019, 0.028, 0.025), and AUC (0.007, 0.015, 0.005) for birds, non-human mammals, and humans, respectively.

Supplementary Figure S7. Combination of risk models in Spain using only spatiotemporal variables from the previous year. Spatiotemporal models for West Nile Virus infection risk in (A) birds, (B) non-human mammals, and (C) humans are combined to identify risk areas based on virus circulation in at least one of these components over the last 20 years (2003–2022). (D) This output is integrated with a model representing areas favorable for the presence of the (E) vector to generate a map that highlights regions with both virus circulation risk and suitable conditions for the vector responsible for its transmission. *I* indicates fuzzy intersection, while U indicate fuzzy union of the models.

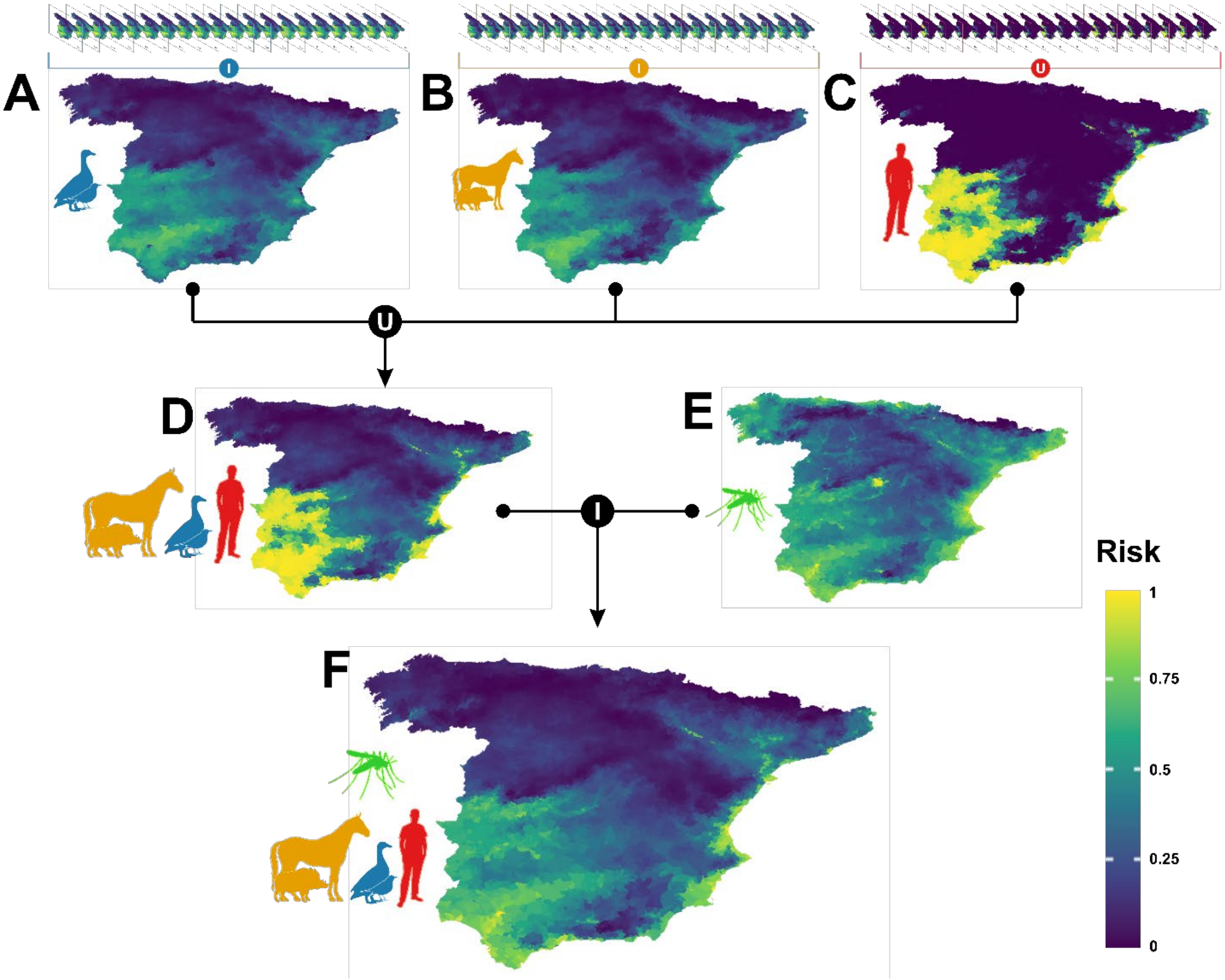

Supplementary Table S6. Predictor variables included in the spatiotemporal models for West Nile virus circulation in birds, mammals, and humans in Spain during the period 2003–2022 considering only spatiotemporal variables (ecosystem and weather) from the previous year. *Estimate* is the value of the coefficient that multiplies the variable value in the logit of the multivariate logistic regression. The *Wald* parameter quantifies the relevance of every variable in the model.

	Bird		Non-human mammal		Human	
**Variable**	**Estimate**	**Wald**	**Estimate**	**Wald**	**Estimate**	**Wald**
Anthropic						
dens_pop	-9.85E-04	1.82E+01	-	-	5.40E-04	4.68E+00
Topo-hydrographic						
altitude	-	-	-1.50E-03	1.91E+01	-	-
slope	-	-	-	-	-4.81E-01	5.57E+00
water-wetness_q	-6.61E-01	4.60E+00	-	-	-	-
Ecosystem						
NDVI mean	-	-	-	-	2.88E-03	5.01E+01
NDVI min	-	-	-	-	-1.76E-03	4.30E+01
NDVI difference	-	-	1.55E-04	8.93E+00	-	-
Weather						
T.mean	-	-	1.15E-01	3.87E+00	1.77E+00	6.89E+01
T.max	2.19E-01	6.69E+01	7.80E-02	1.17E+01	2.42E-01	1.22E+01
P	-	-	-	-	-5.05E-03	1.83E+01
P min	-	-	-4.84E-02	6.73E+00	-	-
P seasonality	-	-	5.11E-03	4.14E+00	3.92E-02	4.57E+01
Zoogeographic						
mosquito	3.14E+00	3.91E+01	-	-	-	-
Intercept	-1.04E+01	1.28E+02	-5.81E+00	3.48E+01	-5.60E+01	1.12E+02
